# Evaluation of Ocular Surface Disease in Asian Patients with Primary Angle Closure

**DOI:** 10.2174/1874364101711010031

**Published:** 2017-02-28

**Authors:** Tan Ee Ling, Khairuddin Othman, Ong Poh Yan, Rasdi Abdul Rashid, Cheong Min Tet, Azhany Yaakob, Liza-Sharmini Ahmad Tajudin

**Affiliations:** 1 Department of Ophthalmology, School of Medical Sciences, Health Campus Universiti Sains Malaysia, 16150 Kubang Kerian, Kelantan, Malaysia; 2 Department of Ophthalmology, Hospital Selayang, Selangor, Malaysia

**Keywords:** Primary angle closure glaucoma, Ocular surface disease, Ocular surface disease index questionnaires, Schirmer’s test, Tear break-up time, Cornea fluorescein staining

## Abstract

**Objective:**

To evaluate the incidence of ocular surface disease (OSD) and to determine the effects of topical pressure-lowering drugs on ocular surface disease in primary angle closure patients.

**Methods:**

This was a cross-sectional comparative study comparing primary angle closure glaucoma (PACG) patients (Group A) with primary angle closure and primary angle closure suspect (Group B). Group A was treated with topical pressure-lowering drugs; Group B was not. Data on ocular diagnosis and details of treatment were obtained from medical records. Ocular surface disease incidence was assessed using the Ocular Surface Disease Index (OSDI) questionnaire and from clinical signs using Schirmer’s test, tear break-up time and corneal fluorescein stain. Predictive Analytic Software 20 and STATA analysis software were used for statistical analyses.

**Results:**

Group A demonstrated a higher rate of OSD (OSDI 52.3%, Schirmer’s test 70.5%, tear break-up time (TBUT) 75%, corneal staining 77.3%) compared to Group B (OSDI 39.0%, Schirmer’s test 73.2%, TBUT 58.5% and cornea staining 14.6%) except for Schirmer’s test. There was a significant difference in mean score of OSDI (p=0.004), TBUT (p=0.008) and cornea staining (p<0.001) between two groups. Primary angle closure glaucoma treated with more than two medications and for more than three years had worse ocular surface disease parameters but without statistical significant difference.

**Conclusion:**

Ocular surface disease is common in PACG patients treated with topical pressure-lowering drugs. Topical pressure-lowering drugs caused significant OSD symptoms and signs except for tear production in PACG patients. Thorough evaluation of ocular surface disease is important to ensure appropriate treatment and intervention in PACG patients.

## INTRODUCTION

Ocular surface disease (OSD) is defined as a group of ocular disorders that affect various component of the ocular surface [1]. OSD is due to increased tear osmolarity, inflammation and disruption of the ocular surface [1, 2]. Risk factors for OSD include the elderly, women especially menopausal women, the Asian population, multiple systemic co-morbidities and ocular diseases [3-8]. Chronic use of topical ophthalmic solutions is another major contributing factor in OSD [9].

Despite the advent of surgical and laser therapies, topical pressure-lowering agents remain the popular choice of treatment in glaucoma. Topical pressure-lowering agents are non-invasive, convenient for patients and easier to discontinue in the presence of side effects or complications [10]. OSD may be induced by topical pressure-lowering agents through chronic toxicity to the preservatives especially benzalkonium chloride (BAK) and active ingredients, alteration of the tear film stability and direct injury to the ocular epithelial surface [11, 12]. The majority of glaucoma patients require multiple medications to achieve the target pressure for a long period, and this further increases the risk for OSD [13-15].

The diagnosis and management of OSD among glaucoma patients are often neglected. The prevalence of OSD among medically treated glaucoma patients was reported to range from 20% to 59% [16, 17]. The majority of the previous studies on OSD were conducted on primary open angle glaucoma (POAG) or ocular hypertensive patients [14, 16, 17]. To the best of our knowledge, there is no study examining OSD in primary angle closure glaucoma (PACG) patients.

Asians are more at risk to develop PACG. Prevalence of PACG has been reported between 0.46% and 1.19% among Asians [26]. PACG among Asians is difficult to manage as compared to primary open angle glaucoma (POAG) [18-20]. Most often multiple medications to achieve the target pressure are needed [18, 21]. Acute angle closure (AAC) in PACG may further increase the risk of OSD due to a sudden spike of intraocular pressure (IOP) and inflammation [22]. Similar to OSD, women are more at risk to develop PACG [3, 4, 23]. Ocular biometry of Asian women predisposed them to develop PACG [24, 25]. Based on these findings, the risk of OSD in PACG patients is postulated to be higher. The main objective of this study was to determine the incidence of OSD based on the signs and symptoms of OSD in Asian patients with primary angle closure suspect (PACS), primary angle closure (PAC) and PACG.

## MATERIAL AND METHODS

A cross-sectional comparative study was conducted involving 85 patients (44 PACG patients and 41 PAC/PACS patients). The study subjects were recruited from the ophthalmology clinic of Hospital Selayang in Selangor and Hospital Universiti Sains Malaysia (HUSM) in Kelantan between May 2012 and March 2014. Ethical approval was received from the human research ethics committee, Universiti Sains Malaysia and the National Medical Research Registry, Malaysia. This study was conducted in accordance with the Declaration of Helsinki for human research.

Subjects were divided into two groups: PACG patients treated with topical pressure-lowering agents (Group A) and PAC and PACS patients who were treated with laser peripheral iridotomy (LPI) but without topical pressure-lowering agents treatment (Group B, control group). The inclusion criteria included a confirmed diagnosis of PACG, PAC and PACS based on the International Society of Geographical and Epidemiological Ophthalmology (ISGEO) [27], age between 40 years old and 75 years old, history of laser peripheral iridotomy (LPI) at least 6 months prior to recruitment and able to understand Ocular Surface Disease Index (OSDI) questionnaires.

Those with a visual acuity worse than 6/60 with the Snellen chart or 1 by the logMAR chart, current or past history of instillation of other topical eye drops other than topical pressure-lowering agents in the past 3 months and a history of previous ocular surgery (including cornea transplant, cataract surgery and glaucoma filtering surgery) were excluded. Patients with a history of other external ocular diseases, *e.g.* meibomianitis, pterygium, and nasolacrimal duct obstruction, were excluded. In addition, those with a previous ocular infection or dry eye and systemic disease with known presentation of dry eye, *e.g.* Sjogren syndrome and other connective tissue diseases, were excluded. Contact lens wearer and patients with punctal plugs were also excluded.

Thorough ocular examinations, including slit-lamp biomicroscopy, gonioscopy, fundus examination and intra-ocular pressure (IOP), were conducted by two investigators (KO and RAR) to determine the eligibility of the patients. The type and number of topical pressure-lowering agents, duration of treatment and presence of AAC were retrieved from patients’ medical records. Duration of the treatment was the duration in months from the initiation of treatment and time of recruitment for this study. The presence of systemic diseases, such as diabetes mellitus, hypertension and hyperlipidaemia, was also documented.

The diagnosis of OSD was based on the scoring of the symptoms and signs of OSDI, which were based on the validated Bahasa Malaysia version of the OSDI questionnaire. Clinical signs were based on the scoring on the Schirmer’s test without anaesthesia, tear break-up time (TBUT) and cornea staining. We selected only one eye per subject for the study; if both eyes were eligible, the right eye was selected.

### OSDI Questionnaires

A trained member of the medical staff was responsible for face-to-face interviews with the subjects. The Bahasa Malaysia version of the OSDI questionnaire consists of 12 items. Each item was graded on a scale of 0 to 4: 0=none of the time; 1=some of the time; 2=half the time; 3=most of the time; and 4=all the time. The total OSDI score is calculated using the following formula:

OSDI= [(sum of the OSDI score) × 100] / [(total number of questions answered) × 4].

### Clinical Evaluation for OSD

The sign for the detection of OSD was conducted by the primary investigator (TEL), who was blinded from the OSDI questionnaire outcome and other clinical data.

### Schirmer’s Test (Without Anaesthesia)

Schirmer’s test was performed in a confined room with the fan or air-conditioner switched off. Topical anaesthesia was not applied during the test. The round bend of the sterile Schirmer’s paper strip (Optitech Eyecare, Allahabad, India) was hooked in the lower cul-de-sac over the junction of the temporal and central one-third of the lower lid margin. The subjects were then asked to gently close their eyelids for 5 minutes before the removal of the strip. The amount of wetting was measured by reading the calibrated scale printed on the paper strip. If the tear front moved unevenly, measurement was made from the notch to the middle of the diagonal line. Abnormal Schirmer’s test is defined as less than 10 mm of wetting after 5 minutes [28]; this is further divided into mild abnormal (8–10 mm of wetting), moderate abnormal (5-7 mm) and severe (less than 5 mm) [28].

### Tear Break-up Time

TBUT is the time measured from when the eyelid is opened to the appearance of the first dry spot formation after the instillation of the fluorescein stain into the inferior cul-de-sac. It is used to measure the distribution of tears on the ocular surface and tear film stability. A fluorescein strip (Bausch & Lomb, Surrey, UK) was used in this clinical test, and sterile normal saline solution was used to wet the fluorescein at the tip of the strip. The stopwatch was used to measure the time. The TBUT was then assessed using a slit lamp (Topcon Corp, Tokyo, Japan) at 10X magnification, using cobalt blue illumination. The fluorescein-stained pre-corneal tear film was observed by moving the slit lamp horizontally at a slow rate from side to side with an unaltered angle between the light and the microscope to identify a black gap or dry spot formation. At the first appearance of a dry spot, the stopwatch was stopped and this was considered as TBUT. The test was repeated two times on each eye, and the mean TBUT was calculated for each eye separately. The normal cut-off value designated in this study was ≥10; 7–9 seconds was considered mild, 5–6 seconds considered moderate and <5 seconds considered severe [29].

### Cornea Staining

After assessing the tear break-up time, the cornea was assessed for any staining. The grading of staining was based on the National Eye Institute system [2]. The amount of staining was graded according to stain intensity: 0, no staining; 1, slight staining; 2, moderate staining; and 3, intense staining.

### Statistical Analysis

Predictive Analytic Software (PASW) 20 and STATA analysis software were used for statistical analyses. The difference in the mean of the OSD parameters between PACG and PAC/PACS was analysed with the independent *t*-test. Comparison between OSD parameters with the number of medications, duration of treatment and presence of AAC was analysed using the Mann–Whitney test. Multivariate linear regression was performed to determine the predictor factors for each OSD parameter.

## RESULTS

A total of 85 subjects (85 eyes) were recruited. There were 44 (51.8%) PACG patients treated with topical pressure-lowering agents (Group A) and 41 (48.2%) angle closure patients (19 PAC and 22 PACS) not treated with topical pressure-lowering agents (Group B). PACG patients (Group A) were older (Table **1**), and there was a 2:1 preponderance of female participants. The PACG patients had significantly poorer visual acuity and a higher IOP at initial presentation (Table **1**). The mean duration since diagnosis of the disease for Group A and B was 5.2(2.7) years and 4.2 (3.8) years respectively (Table **1**). Half (50%) of the PACG patients required three or more anti-glaucoma medication (Table **1**).

The incidence of signs and symptoms of OSD was higher in Group A compared to Group B except for Schirmer’s test. More than half (52.3%) of the PACG patients reported abnormal OSDI scores. In general, more than two-third of the PACG patients demonstrated abnormal Schirmer’s test (70.5%), TBUT (75%) and cornea fluorescein staining (77.3%). Normal OSDI score and normal cornea staining were observed in 61% and 85.4% of patients in group B respectively. However, more than half of them (Group B) demonstrated abnormal TBUT (58.5%) and Schirmer’s test (73.2%). Patients in Group A demonstrated statistically higher OSDI scores, lower TBUT and cornea fluorescein staining compared to Group B (Table **2**). However, there was no significant difference in Schirmer’s test between the two groups (Table **2**).

In general, PACG patients who were treated with more than two topical pressure-lowering agents demonstrated shorter duration in TBUT, less tears production (Schirmer’s test) and abnormal corneal staining but without statistically significant difference except for corneal staining (Table **3**). PACG patients treated with more than two topical pressure-lowering agents had statistically worse positive cornea fluorescein staining (Table **3**). However, they demonstrated better OSDI score than those treated with less or equal to two medications (Table **3**).

The mean duration of treatment in this study was 5.2 ± 2.7 years; PACG patients treated longer than 3 years had worse OSD parameters except on the Schirmer’s test, but these did not reach statistical significance. In multivariate linear regression, visual acuity showed an exponential linear relationship with the mean OSDI score (Table **4**). Age had a negative association with Schirmer’s test and mean TBUT but showed a positive linear association with the cornea staining test (Table **4**). The number of topical pressure-lowering agents had a strong positive linear association with both TBUT and cornea staining.

## DISCUSSION

There was a high incidence (52.3%) of OSD based on symptoms and signs in PACG patients treated with topical pressure-lowering agents; however, this was comparable with reported studies on POAG and ocular hypertension patients [16, 17]. The rate of OSD in angle closure patients (PACS and PAC) was also found to be higher than the general population by indirect comparison [30-32]. To the best of our knowledge, this is the first study specifically reporting OSD in angle closure patients. The high percentage of OSD is not surprising as the majority of recruited angle closure patients were older women at postmenopausal age group (mean age 63.4 ± 7.1 years), which further increased their susceptibility to OSD [33]. Moreover, this present study involved the Asian population who are more susceptible to develop OSD [30].

We also included angle closure patients with a history of AAC attack. AAC with a sudden spike of IOP may affect the ocular surface [22]. Systemic diseases, such as diabetes, hypertension and hyperlipidaemia commonly co-existed in glaucoma patients [34-36]. These systemic diseases and their treatment can directly or indirectly affect the ocular surface, causing dry eyes diseases [37]. It is possible that the high prevalence of OSD that we found was influenced by the presence of systemic co-morbidities; however, excluding them would be impossible, especially in elderly subjects.

In this study, we determined the proportion of OSD based on the presence of clinical signs and symptoms. The OSDI is used to assess the symptoms or subjective measurement of dry eye [32, 38, 39]; it rapidly evaluates OSD to provide a valid and reliable score that reflects good sensitivity and specificity [38]. However, the OSDI questionnaire assesses vision-related quality of life [40]. Thus, we excluded patients with visual acuity worse than logMar 1. As expected, visual acuity of PACG patients was significantly poorer than PACS/PAC patients. Visual acuity may affect the OSDI score, giving rise to falsely abnormal scores in PACG. The score of the OSDI is also affected by pain tolerance; women are known to have lower pain tolerance than men [41, 42].

The percentage of abnormal OSD signs in the present study was higher compared to the findings of Ghosh *et al.* [57], who studied the presence of OSD signs in various types of glaucoma. In our study, the percentage of abnormal TBUT and cornea staining was significantly higher in the PACG group. However, the percentage of abnormal Schirmer’s tests was almost equal in both the PACG and control groups. Perhaps, lipid and mucin layers were more affected in the PACG patients treated with topical pressure-lowering drugs [43, 44].

The aqueous layer is known to be commonly affected in OSD [45]. Schirmer’s test has been reported to have less specificity in the diagnosis of dry eye disease [46]. Furthermore, the test is easily affected by reflex tearing due to discomfort during the procedure and delayed removal of the strip [40, 47], and these may cause an incorrect high value in both the PACG and control groups. In general, there was a lack of association between the signs and symptoms of OSD in the present study. Similar findings were also observed in different populations [16, 47]. Evidence of signs of ocular surface damage may be present despite the absence of OSD symptoms [48]. In the current study, cornea staining correlated best with the ocular surface symptoms, which was in agreement with the findings of Sullivan and associates [47]. This could have been due to a lack of specificity of the other tests and the variation of value of the tests [46, 47].

The number of topical pressure-lowering drugs and duration of treatment are associated with OSD in glaucoma patients [48, 49]. In the present study, number of medications and duration of treatment were not associated with abnormal signs and symptoms of OSD except for corneal staining. In contrast, there was evidence of a significant association between the mean OSDI score and the number of topical pressure-lowering drugs [17]. Histologically, there was evidence of subclinical inflammation in the conjunctival of glaucoma patients treated with topical pressure-lowering drugs [50, 51], but subclinical inflammation is not manifested clinically, which perhaps provides an explanation for our findings. Moreover, histo-morphological changes on the ocular surface epithelium can appear as early as 2 weeks after the commencement of medications [52].

Multiple medications reduce tear secretion, tear stability and induce more cornea epithelial injury [53], which is what we showed even though our findings were not statistically significant. Detrimental effects of topical pressure-lowering drugs on the ocular surface have been shown in normal healthy subjects after a short duration of exposure [54]. A higher prevalence of OSD was expected in patients treated for longer duration. Multiple topical pressure-lowering drugs and a longer duration of treatment may also cause cornea hypesthesia, leading to an underestimation of OSD symptoms [55]. However, duration has less of an effect on changes on the ocular surface. A significant reduction of goblet cells in conjunctiva was found in patients treated with topical pressure-lowering drugs for 3 years or more [51]; in contrast, there was no significant difference in the number of goblet cells between glaucoma patients treated ≥1 year and <1 year [56]. These supported our findings that there was no significant difference in OSD signs between PACG patients treated with topical pressure-lowering drugs for >3years and ≤3 years. A major limitation in the sub-analysis of the effect of topical pressure drugs on the ocular surface of angle closure in this study was the relatively small sample size. The effect of topical pressure-lowering drugs on changes on the ocular surface is still inconclusive.

## CONCLUSION

OSD is common in angle closure patients. A higher incidence was observed in PACG patients treated with topical pressure-lowering drugs, and there were significant severe OSD symptoms and cornea fluorescein staining in PACG patients. It is important to evaluate OSD during the management of angle closure patients, especially those with PACG.

## Figures and Tables

**Table 1:** Demographic and general ophthalmic characteristics of Group A and Group B.

**Clinical characteristic**	**Group A** **(n=44)**	**Group B** **(n=41)**	**p value**
**Age, years (SD)**	63.1 (7.0)	62.8 (6.4)	0.833^
**Sex (n,%)** Female Male	33 (75.0) 11 (25.0)	25 (61.0) 16 (39.0)	0.165*
**Ethnicity (n,%)** Malay Chinese	29 (65.9) 15 (34.1)	24 (58.5) 17 (41.5)	0.483*
**Systemic Diseases (n,%)** Hypertension Diabetes Mellitus Hyperlipidaemia	17 (38.6) 12 (27.2) 9 (20.4)	21 (51.2) 13 (31.7) 11 (34.1)	0.244* 0.654* 0.489*
**Visual Acuity, Log Mar (SD)**	0.36 (0.29)	0.21 (0.23)	**0.012^**
**Intraocular Pressure (at diagnosis),** **mmHg (SD)**	43.6 (8.9)	28.0 (14.4)	**<0.001^**
**Duration of diagnosis, years**	5.2 (2.7)	4.2 (3.8)	0.194^
**Number of medications (n,%)** Monotherapy Dual therapy Triple therapy Quadruple therapy	14 (31.8) 8 (18.2) 13 (29.5) 9 (20.5)	- - - -	0.807*

p<0.005 (*Pearson chi square, ^ independent *t*-test).

**Table 2:** Comparison of the mean score of OSDI, Schirmer’s test, TBUT and cornea staining between Group A and Group B.

**OSD parameters**	**Group A** **(n=44)** **Mean (SD)**	**Group B** **(n= 41)** **Mean (SD)**	**p value**
**OSDI score** **Schirmer’s test (mm)** **TBUT (sec)** **Cornea staining**	19.6 (16.4) 8.6 (7.9) 7.0 (2.6) 5.6 (3.1)	10.4 (12.0) 9.4 (6.8) 8.7 (3.0) 1.7(2.3)	**0.004** 0.639 **0.008** **<0.001**

p<0.05 (independent *t-*test). Abbreviation: OSD; ocular surface disease, OSDI; ocular surface disease index, TBUT; tear break-up time.

**Table 3:** Mean difference in clinical parameters in primary angle closure glaucoma (PACG) patients in association with the number of topical pressure-lowering agents and duration of treatment.

**OSD parameter (mean, SD)**	**Number of medication**		**p value**	**Duration of treatment**		**p value**
	**≤ 2 (N=22)** **Mean (SD)**	**≥ 2 (N=22)** **Mean (SD)**		**≤ 3 years (N=15)** **Mean (SD)**	**>3 years (N=29)** **Mean (SD)**	
**OSDI**	20.5 (17.9)	18.6 (15.1)	0.972	17.3 (16.1)	20.2 (16.7)	0.413
**Schirmers, mm**	9.0 (8.0)	8.3 (8.0)	0.796	6.9 (7.1)	9.6 (8.3)	0.168
**TBUT, seconds**	7.5 (3.0)	6.5 (2.0)	0.311	7.1 (2.6)	7.0 (2.6)	0.940
**Cornea staining**	4.6 (3.1)	6.7 (2.7)	**0.027**	5.3 (2.8)	5.9 (3.2)	0.486

p<0.05 (Mann–Whitney test). Abbreviation: OSD; ocular surface disease, OSDI; ocular surface disease index, TBUT; tear break-up time.

**Table 4:** Multivariate analysis of OSD parameters.

**OSD parameters**	**Multivariate analysis**			
	**Predictors**	**beta**	**95% CI** **(UCI, LCI)**	**p value**
**OSDI** **Schirmer’s test** **TBUT** **Cornea staining**	Visual acuity Age Age Number of treatments Age Number of treatments	17.30 –0.30 –0.09 –0.66 0.10 1.22	(5.77, 28.85) (0.53, 0.06) (–0.18,–0.00) (–1.06,–0.25) (0.02, 0.18) (0.80, 1.65)	**0.004** **0.014** **0.048** **0.002** **0.016** **<0.001**

Backward method applied. The model fits reasonably well. Model assumptions were met. No interaction and multicollinearity problem. Abbreviation: OSD; ocular surface disease, OSDI; ocular surface disease index, TBUT; tear break-up time.

## LIST OF ABBREVIATIONS

TEL: = Tan Ee Ling
KO: = Khairuddin Othman
RAR: = Rasdi Abdul Rashid
